# Cognitive Function Mediates the Anti-suicide Effect of Repeated Intravenous Ketamine in Adult Patients With Suicidal Ideation

**DOI:** 10.3389/fpsyt.2022.779326

**Published:** 2022-05-02

**Authors:** Yanling Zhou, Chengyu Wang, Xiaofeng Lan, Weicheng Li, Ziyuan Chao, Kai Wu, Roger S. McIntyre, Yuping Ning

**Affiliations:** ^1^Department of Psychiatry, The Affiliated Brain Hospital of Guangzhou Medical University (Guangzhou Huiai Hospital), Guangzhou, China; ^2^Guangdong Engineering Technology Research Center for Translational Medicine of Mental Disorders, Guangzhou, China; ^3^School of Biomedical Sciences and Engineering, South China University of Technology, Guangzhou, China; ^4^Canadian Rapid Treatment Center of Excellence, Mississauga, ON, Canada; ^5^Mood Disorders Psychopharmacology Unit, Poul Hansen Depression Centre, University Health Network, Toronto, ON, Canada; ^6^Department of Psychiatry, University of Toronto, Toronto, ON, Canada; ^7^Institute of Medical Science, University of Toronto, Toronto, ON, Canada; ^8^Brain and Cognition Discovery Foundation, Toronto, ON, Canada; ^9^Department of Pharmacology, University of Toronto, Toronto, ON, Canada; ^10^The First School of Clinical Medicine, Southern Medical University, Guangzhou, China

**Keywords:** suicidal ideation, ketamine, depression, processing speed, cognition

## Abstract

**Objective:**

Prior research has shown that ketamine has anti-suicide effects. Additional evidence also suggests that ketamine may offer pro-cognitive effects. Herein, we propose that the anti-suicide effects of ketamine are partially mediated *via* pro-cognitive effects. We aimed to determine whether improvement in cognitive function mediated change in suicidal ideation was associated with ketamine treatment.

**Methods:**

Unipolar or bipolar depressive patients (*n* = 86) with suicidal ideation received six infusions of ketamine (0.5 mg/kg) over 2 weeks. The current severity of suicidal ideation and depression symptoms were assessed with the Beck Scale for Suicide Ideation (SSI) and the Montgomery–Asberg Depression Rating Scale (MADRS), respectively, at baseline, days 13 and 26. Cognitive domains, including processing speed, working memory, visual learning, and verbal learning were measured with the Measurement and Treatment Research to Improve Cognition in Schizophrenia (MATRICS) Consensus Cognitive Battery at the same time points.

**Results:**

Mediation analysis showed a significant total effect of ketamine treatment on SSI score (coef = –1.853, 95%CI [–2.2, –1.5]). The direct and total indirect (MADRS total score and any of cognitive domains) effects of ketamine on suicidal ideation both were statistically significant (direct: coef = –1.064 to –1.352; total indirect: coef = –0.501 to –0.788). MADRS total score and processing speed (but not other cognitive domains) were significant partial mediators of the association between ketamine treatment and improvements in suicidal ideation.

**Conclusion:**

Depressive symptoms severity and processing speed performance partially mediated improvements in suicidal ideation after repeated ketamine infusions in persons with unipolar or bipolar depressive disorder.

## Introduction

Suicide is a serious mental health problem with the number of global suicide mortality of over 800,000 per year and suicide attempts of around 16 million per year (1). Mental disorders are the most frequent diagnoses of suicidal behavior, notably depression (2). Currently, treatment options to improve aspects of suicidality are limited. Conventional interventions including antidepressants, cognitive behavioral therapy, and electroconvulsive therapy, are reported to reduce suicidal ideation and behavior in persons with depression, but this effect takes weeks, leaving a considerable proportion of patients suffering from suicide risk for a long time (3, 4). Moreover, only lithium has demonstrated the ability to lower suicide completion in persons with mood disorders (5).

Fortunately, in the past two decades, numerous RCTs have confirmed ketamine, a glutamate N-methyl-D-aspartate (NMDA) receptor antagonist, has rapid-onset antidepressant effects (6–10). Replicated evidence also indicates that ketamine can rapidly reduce aspects of suicidality (11–17). Suicidal ideation reductions typically manifest within a few hours, and some patients respond as soon as 40 min after the initial infusion (11–17). Currently, nasal esketamine, the S-enantiomer of ketamine has been approved by Food and Drug Administration for the treatment of depressive symptoms in adults with major depressive disorder (MDD) with acute suicidal ideation or behavior (18–20).

Most clinical studies showed a significant decrease in suicidal ideation and overall depressive symptom severity simultaneously following ketamine treatment, suggesting the ketamine’s antisuicide effects are, to a certain extent, mediated by the improvements in overall depressive symptom severity (11, 14). However, Ionescu et al. in an open-label ketamine trial observed a significant decrease in suicidal ideation following six ketamine infusions. The anti-suicide effects approached significance after controlling for total depressive symptom severity, indicating the ketamine’s anti-suicide effects were independent of the antidepressant effects (13). There is a need to determine whether ketamine’s anti-suicide effects are dissociable from its antidepressive effects.

In addition, cognitive dysfunction is recognized a core symptom in depression (21), and the potential pro-cognitive effects in the domains of working memory, visual learning memory, and processing speed with ketamine in treatment-resistant depression (TRD) is suggested by the recent reports (22–25). Our previous study assessed the cognitive effect of six infusions of ketamine in adults with TRD and/or suicidal ideation, and simple improvements in processing speed and verbal learning were observed 1 day following six infusions of ketamine (23). Currently, studies have suggested a close association between vulnerability to suicidal behavior and cognitive alterations (26–28). Deficits in executive function, working memory, decision-making, and impulsivity were associated with current or/and histories of suicidal ideation/behavior in patients with MDD (26–28). Altered value-based and cognitive control processes may be important factors of suicidal vulnerability (29). Available evidence suggests underlying the suicidal processing and help to identify the target of therapeutic interventions aimed at reducing the long-term risk of suicidal acts.

Relatively few studies have evaluated the mediational role of cognition in suicidality in persons with mood disorders. We observed previously that improvements in working memory were associated with anti-suicide outcomes after repeated ketamine infusions in depressed patients with baseline suicidal ideation (30). The foregoing observation supports the previous hypothesis that the anti-suicide effects of ketamine are mediated, in part *via* pro-cognitive effects (30).

However, our previous study takes no account of the effect of improvements in depressive symptoms on the anti-suicide effects following ketamine treatment and lacks the result of other cognitive domains except working memory. Herein, we sought to determine whether improvements in depressive symptoms and/or cognition mediated change in suicidal ideation in adult patients received six sub-anesthetic doses of ketamine, and examine predictors of ketamine’s anti-suicide effects.

## Materials and Methods

### Participants and Study Design

The present data were obtained from a larger single-center open label study comprising a total of 136 adults patients with MDD or bipolar depressive disorder received six sub-anesthetic doses of ketamine (23, 31). This study was approved by the Clinical Research Ethics Committee of the Affiliated Brain Hospital of Guangzhou Medical University and is registered on Chinese Clinical Trial Registry under the identifier ChiCTR-OOC-17012239.

Eligibility criteria and treatment protocol have been outlined previously (23, 31). Briefly, all the subjects were adults (age ≥ 18 years) male or female with a diagnosis of MDD or bipolar disorder supported by the Diagnostic and Statistical Manual of Mental Disorders (DSM-5.0) criteria; moderate-to-severe depressive symptom severity assessed by the 17-item Hamilton Depression Rating Scale (HAMD-17, total scores ≥ 17); and with a suicidal tendency confirmed by a Beck Scale for Suicide Ideation (SSI) part I score ≥ 2 at screening. Participants were excluded if they had a presence of alcohol or substance dependence or any serious or unstable medical conditions identified through physical examination, vital signs, weight, electrocardiogram, blood tests, and urinalysis. Participants were also excluded if had psychotic symptoms or bipolar depressive patients had a mania or hypomania episode in the preceding 6 months.

All the participants received six infusions of intravenous ketamine (0.5 mg/kg) over 2 weeks (infusion on Monday-Wednesday-Friday). Ketamine was administered as an adjunctive to current psychotropic medications which were required to maintain the same dose during the 2-week period. Ketamine hydrochloride injection mixed with 0.9% sodium chloride injections was administered by IV pump over 40 min by a study physician and research nurse. Vital signs (blood pressure, pulse, and oxygen saturation) were monitored throughout the infusion and post-infusion to ensure a return to pre-infusion levels. Additional detailed information regarding these participants has been described in our previous studies (23, 31).

### Measurements

Depressive symptoms, suicidal ideation, and cognitive function were assessed at baseline, 1 day following the sixth infusion (i.e., day 13), and again 14 days following the sixth infusion (i.e., day 26). The Montgomery–Asberg Depression Rating Scale (MADRS) was used to characterize depressive symptoms by clinicians. The total scores range 0–60 and higher scores indicated more severe depressive symptom (32). Current severity of suicidal ideation was assessed *via* the SSI part I, a 5-item scale rating from 0 (least severe) to 2 (most severe) and score range 0–10 (33). Cognitive function was assessed with the Measurement and Treatment Research to Improve Cognition in Schizophrenia (MATRICS) Consensus Cognitive Battery (MCCB) (34). There were seven cognitive domains in the MCCB, but four of them, including processing speed [using the Category Fluency test, Trail Making A test, and Brief Assessment of Cognition in Schizophrenia (BACS)], working memory [using WAIS-III, letter–number sequencing (LNS) subtest, WMS-III, Spatial Span], visual learning (using Brief Visuospatial Memory Test-Revised, BVMT-R), and verbal learning (using Hopkins Verbal Learning Test-Revised, HVLT-R) were selected in the study. Each domain score was standardized to a T score with a mean of 50 and a standard deviation of 10.

### Statistical Analyses

Data were analyzed with IBM Statistical Package for the Social Sciences (SPSS) statistical software for Windows, version 22. All the tests were two-sided with statistical significance at *p* < 0.05. Change in MADRS total score, SSI score, and the four cognitive domain performance following ketamine treatment was assessed using a linear mixed model with measurement point (i.e., baseline, days 13 and 26) as factors. The patients’ characteristics including age, gender, education, duration of illness, body mass index (BMI), and concomitant medications (combined use of mood stabilizer/benzodiazepine/antipsychotic or not, and use of one or two antidepressants) were included as covariates.

Then, mediation analysis using the process v2.15 in SPSS was used to investigate whether overall depressive symptom and cognitive function mediated the anti-suicide effect in patients following six ketamine infusions. In this model measurement point (i.e., baseline, days 13 and 26) was the independent variable (X), anti-suicide effect (i.e., SSI score) was the dependent variable (Y), depressive symptom severity (i.e., MADRS total score) was the first mediator (M1), and cognitive function (i.e., processing speed, working memory, visual learning, verbal learning T score) was the second mediator (M2). Patients’ characteristics, including age, gender, education, duration of illness, BMI, primary diagnosis, and concomitant medications (combined use of mood stabilizer/benzodiazepine/antipsychotic or not, and use of one or two antidepressants) were treated as covariates. Unstandardized beta coefficients (Coef) and *P*-value are reported.

Finally, a binomial logistic regression using forward LR method was completed to determine the effect of baseline cognitive function, depressive symptom severity, and suicidal ideation on the likelihood patients would achieve responder/remitter outcome to six infusions of ketamine, controlling of the foregoing patients’ characteristics. Responders were defined as a 50% or greater decrease in SSI score compared with baseline. Remitters were classified as patients who had a score of zero on the SSI at day 13.

## Results

### Demographic and Clinical Characteristics

The project included 104 MDD or bipolar depression with suicidal ideation received ketamine treatment, and 86 of them received six infusions and completed MADRS, SSI, and MCCB at baseline and day 13 whose data were included in this study. In total, 11.6% of them (*N* = 10) failed to complete the final visit (day 26). Demographic and clinical characteristics are reported in Table 1.

**TABLE 1:** Baseline demographic characteristics of patients (*n* = 86).

Variables	*N*	%
Gender (male)	39	45.3
Employment status (working)	46	53.5
Smoking	16	18.6
Diagnosis (MDD)	66	76.7
Psychiatric comorbidity (yes)^➀^	14	16.3
Having family history of psychiatric disorders	31	36.0
Previous hospitalization (yes)^➁^	25	29.1
Current pharmacotherapies		
At least 1 antidepressant^➂^	86	100
≥ 2 antidepressant	16	18.6
Mood stabilizer^➃^	25	29.1
Benzodiazepine^➄^	40	46.5
Antipsychotic^➅^	45	52.3
	**Mean**	***SD***
Age (years)	33.5	11.2
Education (years)	12.6	3.2
Duration of illness (months)	102.5	89.3
Body mass index (kg/m^2^)	22.6	3.5
Dose of antidepressant (mg/day)^➆^	38.1	21.0

*MDD, Major Depressive Disorder.*

➀*Comorbidity of an Axis I anxiety disorder, obsessive-compulsive disorder, phobia, or panic disorder.*

➁*Previous hospitalization due to mental health problems.*

➂*25 Escitalopram (10–20 mg/day), 12 Duloxetine (60–120 mg/day), 5 Fluvoxamine (150–300 mg/day), 6 Fluoxetine (20–40 mg/day), 11 Paroxetine (20–60 mg/day), 11 Sertraline (100–200 mg/day), 16 Venlafaxine (75–300 mg/day).*

➃*6 Lithium carbonate (300–1,200 mg/day), 7 Lamotrigine (25–250 mg/day), 12 Valproate (1,000–1,500 mg/day).*

➄*12 Lorazepam (1–4 mg/day), 9 Alprazolam (0.2–0.8 mg/day), 14 Oxazepam (15–30 mg/day), 5 Estazolam (1–2 mg/day).*

➅*20 Olanzapine (2.5–20 mg/day), 11 Quetiapine (100–800 mg/day), 2 Risperidone (4 mg/day), 10 Ziprasidone (40–80 mg/day), 2 Amisulpride (600 mg/day).*

➆*Fluoxetine equivalent dose.*

### Mixed Model and Mediation Results

**FIGURE 1:**
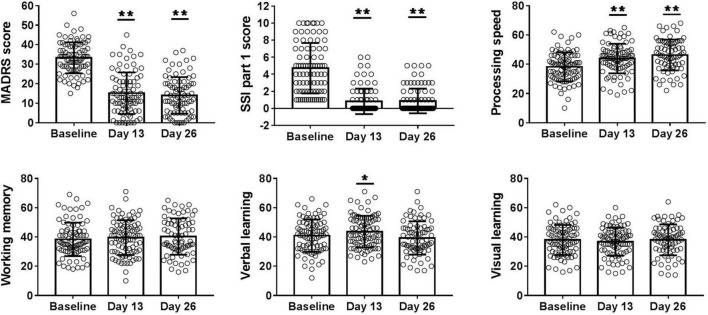
Overall change in MADRS score, SSI part 1 score, T score of processing speed, working memory, visual learning, and verbal learning from baseline to days 13 and 26. A main effect of ketamine treatment was observed in depressive symptoms, suicide ideation, processing speed, and verbal learning performance. MADRS, Montgomery-Asberg Depression Rating Scale; SSI part 1, the first five items of Beck Scale for Suicide Ideation. *P < 0.05, **P < 0.001. All *p*-values represent the results of retrospective analyses compared with baseline.

Six infusions of ketamine were significantly associated with reductions in SSI score and MADRS total score, which have been reported previously. There was a significant main effect of ketamine treatment on SSI score (baseline: 4.7 ± 3.0; day 13: 0.9 ± 1.9; day 26: 1.0 ± 1.9; *F* = 79.643, *P* < 0.001; Figure 1), indicating that six infusions of ketamine were significantly associated with improvement in suicide ideation. The mean MADRS score significantly decreased from baseline (33.2 ± 7.9) to day 13 (15.2 ± 10.6) and day 26 (14.0 ± 9.4), denoting significant improvement in depressive symptom (*F* = 173.769, *P* < 0.001; Figure 1).

Overall change in cognitive function across six infusions of ketamine is presented in Figure 1. The mean T score of processing speed (baseline: 38.4 ± 10.4; day 13: 44.1 ± 10.8; day 26: 46.1 ± 12.1; *F* = 28.537, *P* < 0.001) and verbal learning (baseline: 40.9 ± 11.0; day 13: 43.0 ± 11.8; day 26: 39.3 ± 11.4; *F* = 4.283, *P* = 0.015) significantly increased from baseline to days 13 and 26, denoting significant improvements in these domains of cognitive performance. However, no significant improvement in working memory (baseline: 38.4 ± 11.5; day 13: 39.5 ± 11.9; day 26: 40.3 ± 12.5; *F* = 1.950, *P* = 0.146) and visual learning (baseline: 37.9 ± 10.3; day 13: 36.7 ± 9.6; day 26: 38.1 ± 10.6; *F* = 0.876, *P* = 0.418) was found in the linear mixed models.

**FIGURE 2:**
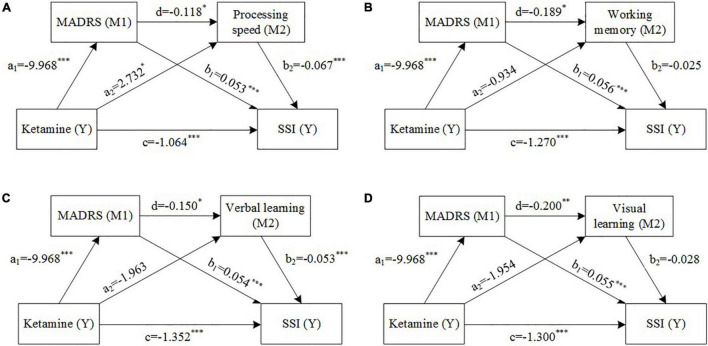
Mediation models with depressive symptom severity (M1) and cognition [M2, **(A)** processing speed, **(B)** working memory, **(C)** visual learning, **(D)** verbal learning] as mediators between ketamine treatment and suicide ideation. Estimated path coefficients are referred to **(A–D)**. *P < 0.05, ^**^P < 0.01, ^***^P < 0.001.

Path analysis was conducted to explore whether improvements in overall depressive symptom severity and cognition acted as the mediator between the ketamine treatment and improvements in suicidal ideation. There was a significant total effect of ketamine treatment on SSI score (coef = –1.853, 95%CI [–2.2, –1.5]). The direct and total indirect effects of ketamine on suicidal ideation both were statistically significant: processing speed as M2 (direct: coef = –1.064, 95%CI [–1.5, –0.6]; total indirect: coef = –0.788, 95%CI [–1.2, –0.4]), working memory as M2 (direct: coef = –1.270, 95%CI [–1.8, –0.8]; total indirect: coef = –0.583, 95%CI [–1.0, –0.2]), verbal learning as M2 (direct: coef = –1.352, 95%CI [–1.8, –0.9]; total indirect: coef = –0.501, 95%CI [–0.9, –0.1]), and visual learning as M2 (direct: coef = –1.300, 95%CI [–1.8, –0.8]; total indirect: coef = –0.552, 95%CI [–0.9, –0.2]). MADRS total score and processing speed T score were significant partial mediators of the relationship between ketamine treatment and improvements in suicidal ideation, however, the foregoing effect was not demonstrated in measures of working memory, verbal learning, or visual learning (Table 2 and Figure 2).

**TABLE 2:** Mediation effects of depressive symptom and cognition on changes in suicidal ideation across treatment.

Parameter	Coef	SE	LLCI	ULCI
Total effect of ketamine on SSI	–1.853	0.202	–2.249	–1.457
**Processing speed was M2**				
Total direct effect of ketamine on SSI	–1.064	0.241	–1.539	–0.590
Total indirect effect of ketamine on SSI	–0.788	0.199	–1.198	–0.399
Ketamine → MADRS → SSI	–0.528	0.176	–0.856	–0.169
Ketamine → MADRS → Processing speed → SSI	–0.078	0.043	–0.168	–0.001
Ketamine → Processing speed → SSI	–0.182	0.091	–0.401	–0.034
**Working memory was M2**				
Total direct effect of ketamine on SSI	–1.270	0.248	–1.758	–0.781
Total indirect effect of ketamine on SSI	–0.583	0.191	–0.976	–0.214
Ketamine → MADRS → SSI	–0.559	0.184	–0.935	–0.196
Ketamine → MADRS → Working memory → SSI	–0.047	0.031	–0.130	–0.005
Ketamine → Working memory → SSI	0.023	0.040	–0.031	0.144
**Verbal learning was M2**				
Total direct effect of ketamine on SSI	–1.352	0.243	–1.831	–0.873
Total indirect effect of ketamine on SSI	–0.501	0.195	–0.882	–0.116
Ketamine → MADRS → SSI	–0.538	0.182	–0.908	–0.201
Ketamine → MADRS → Verbal learning → SSI	–0.068	0.040	–0.160	–0.008
Ketamine → Verbal learning → SSI	0.105	0.070	–0.001	0.267
**Visual learning was M2**				
Total direct effect of ketamine on SSI	–1.300	0.250	–1.792	–0.809
Total indirect effect of ketamine on SSI	–0.552	0.187	–0.908	–0.191
Ketamine → MADRS → SSI	–0.551	0.184	–0.895	–0.193
Ketamine → MADRS → Visual learning → SSI	–0.055	0.035	–0.146	–0.001
Ketamine → Visual learning → SSI	0.054	0.041	–0.003	0.177

*MADRS, Montgomery-Asberg Depression Rating Scale; SSI, Beck Scale for Suicide Ideation; M2, the second mediator.*

### Binomial Logistic Regression Results

The response rate on the SSI at day 13 was 80.2%, and remission rate was 64.0%. In the binomial logistic regression model of responder outcome, when the four baseline cognitive domains were included as independent variables, baseline speed processing was the only variable that predicted achieving an anti-suicide response to six infusions of ketamine (*B* = 0.062, *P* = 0.028). When baseline MADRS score and SSI score, as well as the four baseline cognitive domains were included as independent variables simultaneously in the responder outcome model, the statistical results did not change, baseline processing speed still was the only significant predictor of the anti-suicide response (*B* = 0.062, *P* = 0.028). In the binomial logistic regression model of remitter outcome, when the four baseline cognitive domains were included as independent variables, only baseline processing speed was a significant predictor of remission (*B* = 0.075, *P* = 0.004). When baseline MADRS score and SSI score, as well as the four baseline cognitive domains were included as independent variables simultaneously in the remitter outcome model, baseline SSI score was a significant predictor of remission (*B* = –0.275, *P* = 0.001), while baseline speed processing was excluded in the model.

## Discussion

Herein, we observed six infusions of ketamine in unipolar or bipolar depressive disorders with suicidal ideation were associated with improved mood, suicidal ideation, and cognitive function (i.e., processing speed, verbal learning). Mediational analysis indicated that the anti-suicide effects were partially mediated by improvements in depressive symptom and processing speed. The mediational effect of working memory, verbal learning, or visual learning on the change in SSI was not statistically significant. Herein, we speculate that for some individuals, ketamine’s anti-suicide effects may be subserved by improvements in processing speed. Baseline processing speed was a significant predictor of anti-suicide effect, indicating greater baseline processing speed performance was associated with an increase likelihood of achieving anti-suicide effect over six ketamine infusions.

Cognitive function in mood disorder can be broadly categorized as cold cognition and hot cognition. The former refers to information processing in the absence of any emotional influence, yet the latter is influenced by emotion (35). Usually, cold cognition is considered to include memory, attention, executive function, working memory while hot cognition includes catastrophic reactions to real and/or perceived slights, anhedonia, suicidal ideation, negativistic rumination, negative recall bias, and disproportionate attention to negative stimuli (36). Over the past years, an increasing number of literatures have established that cognitive deficits are closely related to suicidality (26–28). In subgroups of individuals with suicidal ideation and behavior represent deficits in cognitive function (i.e., executive function, impulsivity, attention) (26–28). A conceptual framework of cognitive function in mood disorder was provided by McIntyre and colleagues in a review indicating implicit suicidal ideation as a domain of hot cognition (35). These give us indication that improvements in cognitive function may be closed to the anti-suicide effect by the antidepressant treatment, with no exception to ketamine.

The hot and cold cognitive functions could benefit from subanesthetic dose of ketamine. Ketamine’s benefit on hot cognition is embodied in the improvement in suicidal ideation, anhedonia, and overall mood symptoms (7, 9, 14, 37). Furthermore, ketamine’s enhancement in positive bias and decline in negative emotional perception in MDD further supports its favorable effect on hot cognition (38). The cold cognition benefit is implicated by improvement in working memory, visual memory, and processing speed after ketamine treatment, although some studies reported it was mediated by depressive symptom improvement (22–25).

Our results suggested that repeated ketamine infusions have a pro-cognitive effect independent of decrease in depression severity. Mediational analysis indicated that the improvement in the processing speed was partly independent of decrease in the MADRS score, and the direct effect (coef = 2.732) was greater than the indirect effect (coef = –0.118 × –9.968 = 1.220). The foregoing is in accordance with the results of two other studies with repeated ketamine infusions. McIntyre et al. reported improvement in Trail Making Test-B, measurement processing speed and executive function, was fully independent of improvement in depressive symptom after four infusions in individuals with MDD and bipolar depression (25). Shiroma et al. (24) observed a mood independent pro-cognitive effect on processing speed, attentional set-shifting and spatial working memory among patients with TRD following six doses of ketamine treatment (24).

The results of mediation analysis suggested that the specific effect of ketamine on suicidal ideation also was positively related to pro-cognitive effect. In addition, the present results showed ketamine’s specific decrease in suicidal ideation was larger in individuals with greater processing speed at baseline. In a randomized controlled trial of a single infusion of ketamine, Price et al. reported patients with higher severity of baseline suicidal cognition manifest greater anti-suicide effects, which were partly mediated by a decrease in non-suicide-related depressive symptom (11).

In a naturalistic follow-up study of participants with suicidal ideation found that better cognitive flexibility, assessed by the Wisconsin Card Sorting Test which also reflected executive function, predicted less suicidal ideation after 6 months (39). Our previous clinical study found early increase in levels of kynurenic acid, an NMDA receptor agonist leading to neuroprotection by elevation of extracellular glutamate, could predict anti-suicide response following six infusions of ketamine treatment (40). This is partially supported that patients who have a higher intrinsic “restorative” capacity of neuronal plasticity may be more likely to benefit from ketamine.

In addition, the neuromechanism of ketamine on brain may be involved in the relationship between its pro-cognitive effects and anti-suicide as well as antidepressant effects. Ketamine as an NMDA receptor agonist has a unique mechanism of action involving glutamate modulation *via* increased NMDA and α-amino- 3-hydroxy-5-methyl-4-isoxazolepropionic acid (AMPA) receptor throughput, leading to potentiation of BDNF and mechanistic target of rapamycin (mTOR) signaling pathways, and these finally contribute to the augmented synaptogenesis and synapse stabilization (41, 42). Ketamine’s glutamatergic modulation of numerous brain regions and neural circuits, as shown in neuroimaging studies, was considered to involve in its antidepressant and anti-suicide action, and also pro-cognitive effect (43–46). The prefrontal cortex (PFC) global connectivity could be normalized in responders and the extent of the PFC global connectivity increase was associated with response after a single dose of ketamine (43, 44). Another study found increased hippocampal volumes and decreased nucleus accumbens volumes 24 h post-single dose of ketamine (45). Our previous study also showed increased volumes of the left amygdale and the right hippocampus after six infusions of ketamine (46). These brain regions and neural circuits correlated to ketamine’s treating response also involve hot and cold cognitive functions in patients with MDD, as mentioned in the previous study (36). Herein, we propose that ketamine’s antidepressant effects are mediated, in part, by targeting neural circuits that subserve the relevant to cognitive processing and emotional processing (35). It stands to reason that given ketamine’s known effect on neural structures and functions, its anti-suicide effects may be in part *via* pro-cognitive effects. The results from this study provide some clues for future work aimed to understand the neural mechanism for the effect of ketamine on suicidal ideation, and these evidences augmented with neuroimaging would be an important pathway for future work.

The present findings should be considered the following limitations. First, the results were obtained from a single arm open label study without a control arm. This may introduce expectation bias both to assessments from the clinician and patients. Second, several lines of evidence suggested that executive function, particularly the decision-making was related to suicidality. Decision-making performance was measured usually using the Iowa Gambling Task (IGT), and poorly performed by patients with current or history of suicide attempt/ideation was observed compared with the healthy controls (28, 29). This study only assessed four cognitive domains, although the Category Fluency test, Trail Making A test, and BACS could well reflect the executive function, lack of decision-making tasks was a significant limitation to describe a complete association between cognition and suicidal ideation. This limitation led to an incomplete relationship between cognitive function and anti-suicide effects of ketamine. Third, patients with severe suicidal ideation or suicidal behavior were excluded for safety reason, therefore, making it difficult to generalize present results to that population. Thus, our findings should not be extrapolated to outcomes of suicidal behavior, particularly attempted or completed suicide. Fourth, all subjects were taking their prior prescribed medications, including benzodiazepine, lithium, and antipsychotics, which may moderate changes in cognition. While changes to medication were not permissible during the study, the effect of psychiatric medication could confuse the observed outcomes. Therefore, the current findings could not be extrapolated to medication-free patients. An additional limitation is we were looking at reduction in suicidal ideations future studies should be looking at reductions in suicide and we do not know if reductions in suicidal ideations would necessarily predict reduction in suicide.

## Conclusion

The present results provide insights into potential moderators and psychological mechanisms of ketamine’s anti-suicide effects, which are associated with pro-cognitive effects. Further study is warranted to explore the mechanisms underpinning ketamine’s anti-suicide effects which might reflect glutamate-mediated synaptogenesis and neural circuits serving cognitive function. Future research should also endeavor to combine ketamine with psychosocial strategies (e.g., cognitive behavioral therapy) to determine whether there’s a more robust improvement in measures of suicidality and cognitive function.

## Data Availability Statement

The raw data supporting the conclusions of this article will be made available by the authors, without undue reservation.

## Ethics Statement

The studies involving human participants were reviewed and approved by the Clinical Research Ethics Committee of The Affiliated Brain Hospital of Guangzhou Medical University. The patients/participants provided their written informed consent to participate in this study. Written informed consent was obtained from the individual(s) for the publication of any potentially identifiable images or data included in this article.

## Author Contributions

YZ, YN, and RM developed research hypothesis and study design. YZ conducted data analysis and wrote the final draft of the manuscript. CW, XL, WL, KW, and ZC involved with data collection. All authors contributed to the final manuscript proofreading, edits, and approval for submission.

## Conflict of Interest

The authors declare that the research was conducted in the absence of any commercial or financial relationships that could be construed as a potential conflict of interest.

## Publisher’s Note

All claims expressed in this article are solely those of the authors and do not necessarily represent those of their affiliated organizations, or those of the publisher, the editors and the reviewers. Any product that may be evaluated in this article, or claim that may be made by its manufacturer, is not guaranteed or endorsed by the publisher.

## References

[1] World Health Organization [WHO]. *Preventing Suicide: A Global Imperative.* Geneva: World Health Organization (2014).

[2] Bachmann S. Epidemiology of suicide and the psychiatric perspective. *Int J Environ Res Public Health.* (2018) 15:1425. 10.3390/ijerph15071425 29986446 PMC6068947

[3] Ballard ED Snider SL Nugent AC Luckenbaugh DA Park L Zarate CA Jr. Active suicidal ideation during clinical antidepressant trials. *Psychiatry Res.* (2017) 257:303–8. 10.1016/j.psychres.2017.07.065 28787656 PMC5626625

[4] D’Anci KE Uhl S Giradi G Martin C. Treatments for the prevention and management of suicide: a systematic review. *Ann Intern Med.* (2019) 171:334–42. 10.7326/M19-0869 31450239

[5] McIntyre RS Berk M Brietzke E Goldstein BI López-Jaramillo C Kessing LV Bipolar disorders. *Lancet.* (2020) 396:1841–56. 10.1016/S0140-6736(20)31544-0 33278937

[6] aan het Rot M Collins KA Murrough JW Perez AM Reich DL Charney DS Safety and efficacy of repeated-dose intravenous ketamine for treatment-resistant depression. *Biol Psychiatry.* (2010) 67:139–45. 10.1016/j.biopsych.2009.08.038 19897179

[7] Murrough JW Iosifescu DV Chang LC Al Jurdi RK Green CE Perez AM Antidepressant efficacy of ketamine in treatment-resistant major depression: a two-site randomized controlled trial. *Am J Psychiatry.* (2013) 170:1134–42. 10.1176/appi.ajp.2013.13030392 23982301 PMC3992936

[8] Murrough JW Perez AM Pillemer S Stern J Parides MK aan het Rot M Rapid and longer-term antidepressant effects of repeated ketamine infusions in treatment-resistant major depression. *Biol Psychiatry.* (2013) 74:250–6. 10.1016/j.biopsych.2012.06.022 22840761 PMC3725185

[9] Singh JB Fedgchin M Daly EJ De Boer P Cooper K Lim P A double-blind, randomized, placebo-controlled, dose-frequency study of intravenous ketamine in patients with treatment-resistant depression. *Am J Psychiatry.* (2016) 173:816–26. 10.1176/appi.ajp.2016.16010037 27056608

[10] McIntyre RS Rosenblat JD Nemeroff CB Sanacora G Murrough JW Berk M Synthesizing the evidence for ketamine and esketamine in treatment-resistant depression: an international expert opinion on the available evidence and implementation. *Am J Psychiatry.* (2021) 178:383–99. 10.1176/appi.ajp.2020.20081251 33726522 PMC9635017

[11] Price RB Iosifescu DV Murrough JW Chang LC Al Jurdi RK Iqbal SZ Effects of ketamine on explicit and implicit suicidal cognition: a randomized controlled trial in treatment-resistant depression. *Depress Anxiety.* (2014) 31:335–43. 10.1002/da.22253 24668760 PMC4112410

[12] Murrough JW Soleimani L DeWilde KE Collins KA Lapidus KA Iacoviello BM Ketamine for rapid reduction of suicidal ideation: a randomized controlled trial. *Psychol Med.* (2015) 45:3571–80. 10.1017/S0033291715001506 26266877

[13] Ionescu DF Swee MB Pavone KJ Taylor N Akeju O Baer L Rapid and sustained reductions in current suicidal ideation following repeated doses of intravenous ketamine: secondary analysis of an open-label study. *J Clin Psychiatry.* (2016) 77:e719–25. 10.4088/JCP.15m10056 27232360

[14] Grunebaum MF Galfalvy HC Choo TH Keilp JG Moitra VK Parris MS Ketamine for rapid reduction of suicidal thoughts in major depression: a midazolam-controlled randomized clinical trial. *Am J Psychiatry.* (2018) 175:327–35. 10.1176/appi.ajp.2017.17060647 29202655 PMC5880701

[15] Zhan Y Zhang B Zhou Y Zheng W Liu W Wang C A preliminary study of anti-suicidal efficacy of repeated ketamine infusions in depression with suicidal ideation. *J Affect Disord.* (2019) 251:205–12. 10.1016/j.jad.2019.03.071 30927581

[16] McIntyre RS Rodrigues NB Lee Y Lipsitz O Subramaniapillai M Gill H The effectiveness of repeated intravenous ketamine on depressive symptoms, suicidal ideation and functional disability in adults with major depressive disorder and bipolar disorder: Results from the Canadian Rapid Treatment Center of Excellence. *J Affect Disord.* (2020) 274:903–10. 10.1016/j.jad.2020.05.088 32664031

[17] Feeney A Hock RS Freeman MP Flynn M Hoeppner B Iosifescu DV The effect of single administration of intravenous ketamine augmentation on suicidal ideation in treatment-resistant unipolar depression: Results from a randomized double-blind study. *Eur Neuropsychopharmacol.* (2021) 49:122–32. 10.1016/j.euroneuro.2021.04.024 34090255 PMC8338746

[18] Canuso CM Singh JB Fedgchin M Alphs L Lane R Lim P Efficacy and safety of intranasal esketamine for the rapid reduction of symptoms of depression and suicidality in patients at imminent risk for suicide: results of a double-blind, randomized, placebo-controlled study. *Am J Psychiatry.* (2018) 175:620–30. 10.1176/appi.focus.17105 29656663

[19] Fu DJ Ionescu DF Li X Lane R Lim P Sanacora G Esketamine nasal spray for rapid reduction of major depressive disorder symptoms in patients who have active suicidal ideation with intent: double-blind, randomized study (ASPIRE I). *J Clin Psychiatry.* (2020) 81:19m13191. 10.4088/JCP.19m13191 32412700

[20] Xiong J Lipsitz O Chen-Li D Rosenblat JD Rodrigues NB Carvalho I The acute antisuicidal effects of single-dose intravenous ketamine and intranasal esketamine in individuals with major depression and bipolar disorders: a systematic review and meta-analysis. *J Psychiatr Res.* (2021) 134:57–68. 10.1016/j.jpsychires.2020.12.038 33360864

[21] McIntyre RS Cha DS Soczynska JK Woldeyohannes HO Gallaugher LA Kudlow P Cognitive deficits and functional outcomes in major depressive disorder: determinants, substrates, and treatment interventions. *Depress Anxiety.* (2013) 30:515–27. 10.1002/da.22063 23468126

[22] Shiroma PR Albott CS Johns B Thuras P Wels J Lim KO. Neurocognitive performance and serial intravenous subanesthetic ketamine in treatment-resistant depression. *Int J Neuropsychopharmacol.* (2014) 17:1805–13. 10.1017/S1461145714001011 24963561

[23] Zhou Y Zheng W Liu W Wang C Zhan Y Li H Neurocognitive effects of six ketamine infusions and the association with antidepressant response in patients with unipolar and bipolar depression. *J Psychopharmacol.* (2018) 32:1118–26. 10.1177/0269881118798614 30260273

[24] Shiroma PR Thuras P Wels J Albott CS Erbes C Tye S Neurocognitive performance of repeated versus single intravenous subanesthetic ketamine in treatment resistant depression. *J Affect Disord.* (2020) 277:470–7. 10.1016/j.jad.2020.08.058 32871534

[25] McIntyre RS Rosenblat JD Rodrigues NB Lipsitz O Chen-Li D Lee JG The effect of intravenous ketamine on cognitive functions in adults with treatment-resistant major depressive or bipolar disorders: Results from the Canadian rapid treatment center of excellence (CRTCE). *Psychiatry Res.* (2021) 302:113993. 10.1016/j.psychres.2021.113993 34034067

[26] Richard-Devantoy S Berlim MT Jollant F. A meta-analysis of neuropsychological markers of vulnerability to suicidal behavior in mood disorders. *Psychol Med.* (2014) 44:1663–73. 10.1017/S0033291713002304 24016405

[27] Richard-Devantoy S Berlim MT Jollant F. Suicidal behaviour and memory: a systematic review and meta-analysis. *World J Biol Psychiatry.* (2015) 16:544–66. 10.3109/15622975.2014.925584 25112792

[28] Alacreu-Crespo A Guillaume S Sénèque M Olié E Courtet P. Cognitive modelling to assess decision-making impairments in patients with current depression and with/without suicide history. *Eur Neuropsychopharmacol.* (2020) 36:50–9. 10.1016/j.euroneuro.2020.04.006 32456851

[29] Richard-Devantoy S Olié E Guillaume S Bechara A Courtet P Jollant F. Distinct alterations in value-based decision-making and cognitive control in suicide attempters: toward a dual neurocognitive model. *J Affect Disord.* (2013) 151:1120–4. 10.1016/j.jad.2013.06.052 23876195

[30] Chen X Wang M Hu Y Zhan Y Zhou Y Zheng W Working memory associated with anti-suicidal ideation effect of repeated-dose intravenous ketamine in depressed patients. *Eur Arch Psychiatry Clin Neurosci.* (2021) 271:431–8. 10.1007/s00406-020-01221-z 33386430

[31] Zheng W Zhou YL Liu WJ Wang CY Zhan YN Li HQ Rapid and longer-term antidepressant effects of repeated-dose intravenous ketamine for patients with unipolar and bipolar depression. *J Psychiatr Res.* (2018) 106:61–8. 10.1016/j.jpsychires.2018.09.013 30278319

[32] Montgomery SA Asberg M. A new depression scale designed to be sensitive to change. *Br J Psychiatry.* (1979) 134:382–9. 10.1192/bjp.134.4.382 444788

[33] Beck AT Kovacs M Weissman A. Assessment of suicidal intention: the scale for suicide ideation. *J Consult Clin Psychol.* (1979) 47:343–52. 10.1037//0022-006x.47.2.343 469082

[34] Nuechterlein KH Green MF Kern RS Baade LE Barch DM Cohen JD The MATRICS Consensus Cognitive Battery, part 1: test selection, reliability, and validity. *Am J Psychiatry.* (2008) 165:203–13. 10.1176/appi.ajp.2007.07010042 18172019

[35] Lee Y Syeda K Maruschak NA Cha DS Mansur RB Wium-Andersen IK A new perspective on the anti-suicide effects with ketamine treatment: a procognitive effect. *J Clin Psychopharmacol.* (2016) 36:50–6. 10.1097/JCP.0000000000000441 26658082

[36] Roiser JP Sahakian BJ. Hot and cold cognition in depression. *CNS Spectr.* (2013) 18:139–49. 10.1017/S1092852913000072 23481353

[37] Lally N Nugent AC Luckenbaugh DA Niciu MJ Roiser JP Zarate CA Jr. Neural correlates of change in major depressive disorder anhedonia following open-label ketamine. *J Psychopharmacol.* (2015) 29:596–607. 10.1177/0269881114568041 25691504 PMC5116382

[38] Murrough JW Collins KA Fields J DeWilde KE Phillips ML Mathew SJ Regulation of neural responses to emotion perception by ketamine in individuals with treatment-resistant major depressive disorder. *Transl Psychiatry.* (2015) 5:e509. 10.1038/tp.2015.10 25689570 PMC4445748

[39] Miranda R Gallagher M Bauchner B Vaysman R Marroquín B. Cognitive inflexibility as a prospective predictor of suicidal ideation among young adults with a suicide attempt history. *Depress Anxiety.* (2012) 29:180–6. 10.1002/da.20915 22147587

[40] Zhou Y Zheng W Liu W Wang C Zhan Y Li H Antidepressant effect of repeated ketamine administration on kynurenine pathway metabolites in patients with unipolar and bipolar depression. *Brain Behav Immun.* (2018) 74:205–12. 10.1016/j.bbi.2018.09.007 30213652

[41] Zanos P Moaddel R Morris PJ Georgiou P Fischell J Elmer GI NMDAR inhibition-independent antidepressant actions of ketamine metabolites. *Nature.* (2016) 533:481–6. 10.1038/nature17998 27144355 PMC4922311

[42] Matveychuk D Thomas RK Swainson J Khullar A MacKay MA Baker GB Ketamine as an antidepressant: overview of its mechanisms of action and potential predictive biomarkers. *Ther Adv Psychopharmacol.* (2020) 10:2045125320916657. 10.1177/2045125320916657 32440333 PMC7225830

[43] Abdallah CG Averill CL Salas R Averill LA Baldwin PR Krystal JH Prefrontal connectivity and glutamate transmission: relevance to depression pathophysiology and ketamine treatment. *Biol Psychiatry Cogn Neurosci Neuroimaging.* (2017) 2:566–74. 10.1016/j.bpsc.2017.04.006 29034354 PMC5635826

[44] Abdallah CG Averill LA Collins KA Geha P Schwartz J Averill C Ketamine treatment and global brain connectivity in major depression. *Neuropsychopharmacology.* (2017) 42:1210–9. 10.1038/npp.2016.186 27604566 PMC5437875

[45] Abdallah CG Jackowski A Salas R Gupta S Sato JR Mao X The nucleus accumbens and ketamine treatment in major depressive disorder. *Neuropsychopharmacology.* (2017) 42:1739–46. 10.1038/npp.2017.49 28272497 PMC5518908

[46] Zhou YL Wu FC Liu WJ Zheng W Wang CY Zhan YN Volumetric changes in subcortical structures following repeated ketamine treatment in patients with major depressive disorder: a longitudinal analysis. *Transl Psychiatry.* (2020) 10:264. 10.1038/s41398-020-00945-9 32747631 PMC7400625

